# Activation and Regulation of B Cell Responses by Invariant Natural Killer T Cells

**DOI:** 10.3389/fimmu.2018.01360

**Published:** 2018-06-18

**Authors:** Derek G. Doherty, Ashanty M. Melo, Ana Moreno-Olivera, Andreas C. Solomos

**Affiliations:** Discipline of Immunology, Trinity Translational Medicine Institute, Trinity College Dublin, Dublin, Ireland

**Keywords:** invariant natural killer T cells, B cells, antibodies, disease, CD1d, glycolipids

## Abstract

CD1d-restricted invariant natural killer T (iNKT) cells play central roles in the activation and regulation of innate and adaptive immunity. Cytokine-mediated and CD1d-dependent interactions between iNKT cells and myeloid and lymphoid cells enable iNKT cells to contribute to the activation of multiple cell types, with important impacts on host immunity to infection and tumors and on the prevention of autoimmunity. Here, we review the mechanisms by which iNKT cells contribute to B cell maturation, antibody and cytokine production, and antigen presentation. Cognate interactions with B cells contribute to the rapid production of antibodies directed against conserved non-protein antigens resulting in rapid but short-lived innate humoral immunity. iNKT cells can also provide non-cognate help for the generation of antibodies directed against protein antigens, by promoting the activation of follicular helper T cells, resulting in long-lasting adaptive humoral immunity and B cell memory. iNKT cells can also regulate humoral immunity by promoting the development of autoreactive B cells into regulatory B cells. Depletions and functional impairments of iNKT cells are found in patients with infectious, autoimmune and malignant diseases associated with altered B cell function and in murine models of these conditions. The adjuvant and regulatory activities that iNKT cells have for B cells makes them attractive therapeutic targets for these diseases.

## Invariant Natural Killer T (iNKT) Cells Control Innate and Adaptive Immune Responses

Invariant natural killer T cells are frequently considered a “bridge” between the innate and adaptive immune systems. They are classed as innate T cells because their T cell receptors (TCRs) are semi-conserved and display specificity for conserved non-peptide antigens. They display effector-memory phenotypes and can respond immediately to infection or inflammation without the need for prior antigen priming. iNKT cells possess multiple effector functions, similar to those of conventional T cells of the adaptive immune system, such as targeted granular release of cytolytic mediators and the release of T helper type 1 (Th1), Th2, Th17, and regulatory (T_reg_) cytokines, allowing them to activate, polarize, and regulate adaptive immune responses. Ultimately, iNKT cell responses can dictate the outcomes of microbial infections, autoimmune diseases, and cancer, and for this reason, they are attractive potential targets for therapeutic intervention for multiple types of disease. However, iNKT cells are more than simply the conjoining cell type linking innate and adaptive immunity. They can stimulate and regulate multiple cell types at many levels and thereby are central controllers of innate and adaptive immune responses.

**Figure 1:**
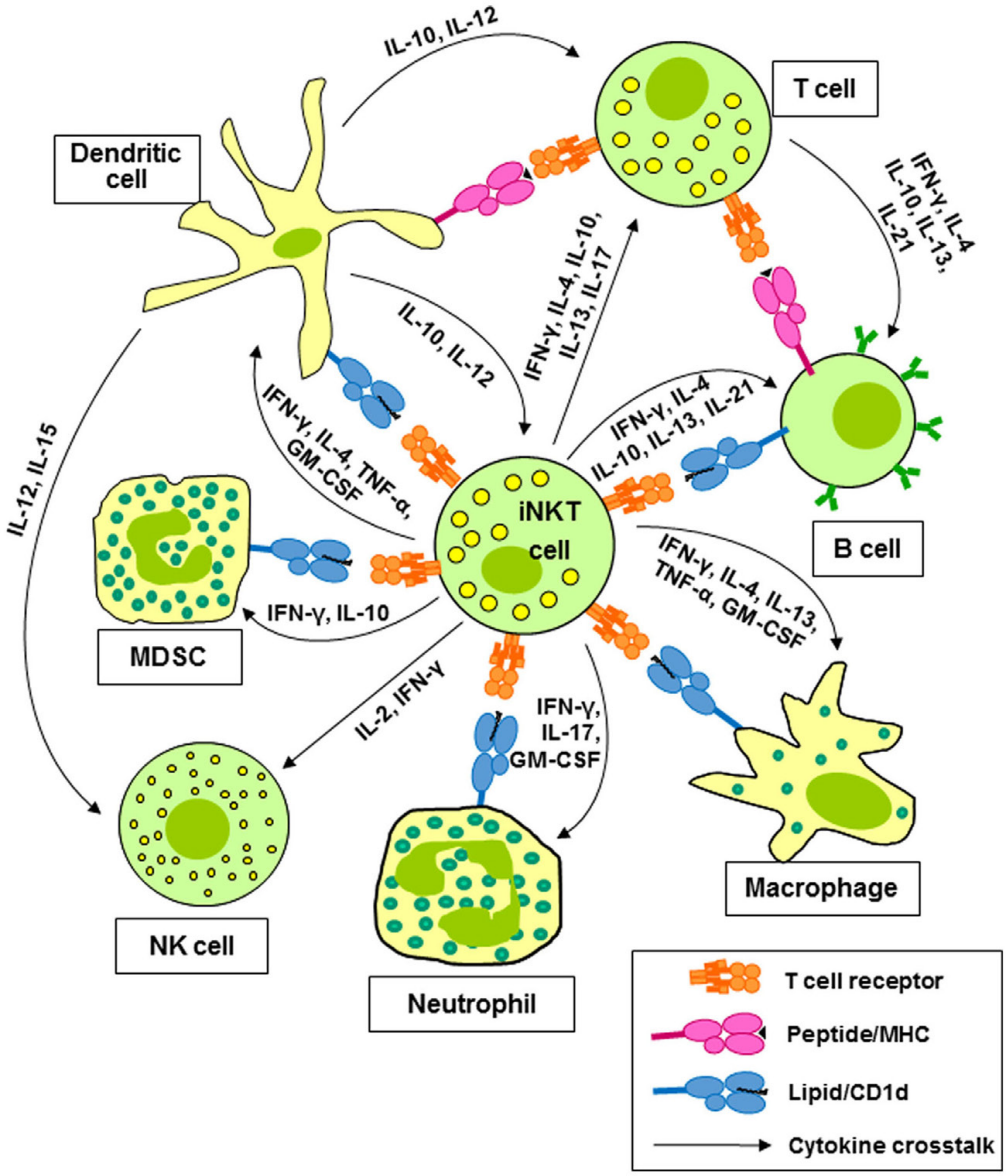
Invariant natural killer T (iNKT) cells activate and regulate multiple cells of the innate and adaptive immune systems. Cytokines released by iNKT cells contribute to the activation and polarization of CD4^+^ and CD8^+^ T cells and natural killer (NK) cells. Cytokines and CD1d-dependent interactions between iNKT cells and dendritic cells (DCs), macrophages, neutrophils, and myeloid-derived suppressor cells (MDSC) lead to the activation and regulation of the effector activities of these cells.

Invariant natural killer T cells, also known as type 1 NKT cells, are clonally expanded T cells expressing a TCR composed of an invariant α-chain (Vα24-Jα18 in human and Vα14-Jα18 in mice) paired with a restricted set of β-chains, which displays specificity for glycolipid antigens presented by CD1d (1, 2). This T cell population is the best characterized member of a wider repertoire of CD1d-restricted T cells, mostly with undefined TCR specificities. CD1d-restricted T cells other than iNKT cells are collectively termed type 2 NKT cells (3, 4). The present review will focus mainly on type 1 NKT cells. Type 1 or iNKT cells express a number of stimulatory receptors that are frequently found on natural killer (NK) cells, such as NK1.1 in mice and NKG2C and NKG2D in humans. Their TCRs can recognize a number of self (5, 6) and microbial (7, 8) glycosphingolipids; however, most research on murine and human iNKT cells has utilized the prototypic glycolipid, α-galactosylceramide (α-GalCer), which binds to CD1d and activates murine and human iNKT cells (9). Activation of iNKT cells with α-GalCer *in vitro* results in target cell killing and the rapid release of multiple growth factors and cytokines (1, 2). iNKT cells are of particular interest because of their ability to produce cytokines associated with all of the CD4^+^ helper T (Th) cell lineages, including the Th1 cytokines interferon-γ (IFN-γ) and tumor necrosis factor-α (TNF-α), the Th2 cytokines interleukin-4 (IL-4), IL-5, and IL-13, the Th9 cytokine IL-9, the Th17 cytokines IL-17A and IL-22, and the T_reg_ cytokine IL-10 (10, 11). These cytokines contribute to the activation and polarization of CD4^+^ and CD8^+^ T cells (12) and natural killer (NK) cells (12, 13). Cytokines and CD1d-dependent interactions between iNKT cells and dendritic cells (DCs) (14, 15), macrophages (16), neutrophils (17, 18), and myeloid-derived suppressor cells (MDSC) (19, 20) lead to the activation and regulation of the effector activities of these cells (Figure 1).

Invariant natural killer T cells make essential contributions to adaptive immune responses by promoting the maturation of dendritic cells into antigen-presenting cells (APCs). Physical interactions between activated iNKT cells and DC result in the expression of major histocompatibility complex (MHC) class II molecules, co-stimulatory molecules such as CD80 and CD86, and the release of IL-12 by the DC. This adjuvant effect involves CD1d/TCR, CD40/CD40L, and CD80/86/CD28 interactions between the two cells (21–24). iNKT cells also promote DC maturation *in vivo*: a single intravenous dose of α-GalCer can stimulate the maturation of DCs into APCs, capable of activating CD4^+^ and CD8^+^ T cells specific for a co-administered protein (25). iNKT cells prime DC to cross-prime CD8^+^ T cells (15, 26, 27) and to promote CD4^+^ Th1 and Th2 cell differentiation and activation (14, 28–30). A subset of iNKT cells can also kill DC (31).

Invariant natural killer T cells can also provide help for B cells. They can boost antibody responses by directly interacting with B cells presenting glycolipid antigens on CD1d (cognate B cell help) and indirectly by activating follicular helper T (T_FH_) cells specific for protein antigens presented by B cells on MHC class II (non-cognate B cell help). This adjuvant activity makes iNKT cells and glycolipids attractive targets for boosting vaccine responses and preventing antibody-mediated diseases. iNKT cells also can regulate pathogenic B cell responses. Here, we review recent findings on the roles and mechanisms by which iNKT cells influence B cell function and how they may contribute to the pathogenesis of and protection against diseases that involve aberrant B cell proliferation, maturation, or regulation.

## Activation of B Cells

B cells are uniquely able to recognize antigens that bind their surface immunoglobulin (Ig) receptors, resulting in the release of soluble antibody, which mediates the humoral immune response through pathogen neutralization, opsonization, and complement fixation. In the adaptive immune system, naïve antigen-specific lymphocytes are rarely activated by antigen alone. Naïve T cell activation requires an antigen-specific signal through the TCR and a co-stimulatory signal from a professional APC. Naïve B cell activation requires antigen recognition by the Ig receptor and additional signals that can come either from a CD4^+^ T cell (thymus-dependent) or, in some cases, directly from microbial components (thymus-independent). B cells and T cells sample antigens in secondary lymphoid tissues, the lymph nodes, and the spleen, which provide a microenvironment that is ideal for enabling physical interactions between T cells and B cells and APCs, such as macrophages and DC. Lymph nodes receive antigens from the tissues *via* the lymphatics. Lymph nodes are divided into lobules, each consisting of an outer B cell-rich cortical region, a T cell-rich paracortical region, and an inner medulla (32). The B cells cluster together in lymphoid follicles. Upon antigenic stimulation, B cells proliferate and form germinal centers, where their Ig genes undergo somatic hypermutation and class switch recombination (33–37). The spleen receives antigens from the blood and consists of the white pulp embedded in red pulp. T cells and B cells accumulate in the white pulp, whereas erythrocytes dominate the red pulp. Murine spleen has an additional B cell-rich area, the marginal zone, between the white and red pulp, a region that is absent in human spleen (38).

Thymus-dependent B cell responses require the dual recognition of antigen by B cells and T cells. DC internalize protein antigens in the tissues and migrate *via* the lymphatics to the T cell-rich zones of lymph nodes. Here, they present antigenic peptides bound to MHC class II molecules to naïve T cells. T cell activation is associated with their differentiation into T_FH_ cells, characterized by the expression of the B cell lymphoma-6 (Bcl-6) transcription factor, CD40 ligand, inducible T cell costimulator, the chemokine receptors CXCR4 and CXCR5, IL-21, programmed death-1 (PD-1), and signaling lymphocytic activation molecule-associated protein (SAP) (33, 39). The T_FH_ cells then relocate to the borders between the T and B cell areas, where they interact with antigen-specific B cells. In the lymph node follicles, the same antigen binds to a B cell receptor (BCR), resulting in internalization, processing, and cell-surface presentation on MHC class II molecules. The B cell migrates to the T–B borders, where it presents antigen to a T_FH_ cells (34–36). Upon recognition of the peptide–MHC class II complex, the T cell expresses CD40 ligand, which ligates CD40 on the B cell, leading to B cell proliferation and differentiation. The T cell also secretes the cytokines IFN-γ, IL-4, IL-10, and IL-21, which are required for Ig isotype switching (40, 41). Cognate T cell–B cell interactions result in the proliferation of B cells in germinal centers, and somatic hypermutation, and affinity maturation of their Igs, resulting in the generation of long-lived antibody-secreting plasma cells and memory B cells (36). Cognate T–B cell interactions can also stimulate extrafollicular proliferation of B cells and their maturation into plasmablasts, which do not undergo affinity maturation and are short-lived. These B cells mediate transient innate-like responses (35).

Thymus-independent B cell responses are elicited by non-protein antigens that do not stimulate T cells, such as bacterial polysaccharides and microbial toll-like-receptor ligands. These antigens are recognized by subsets of innate B cells, such as marginal zone B cells and B-1 cells, which do not reside in the follicles. Thymus-independent responses generally lead to rapid antibody responses to pathogens mediated by short-lived, low-affinity extrafollicular plasma cells (42, 43).

## iNKT Cells Can Provide Non-Cognate B Cell Help

**Figure 2:**
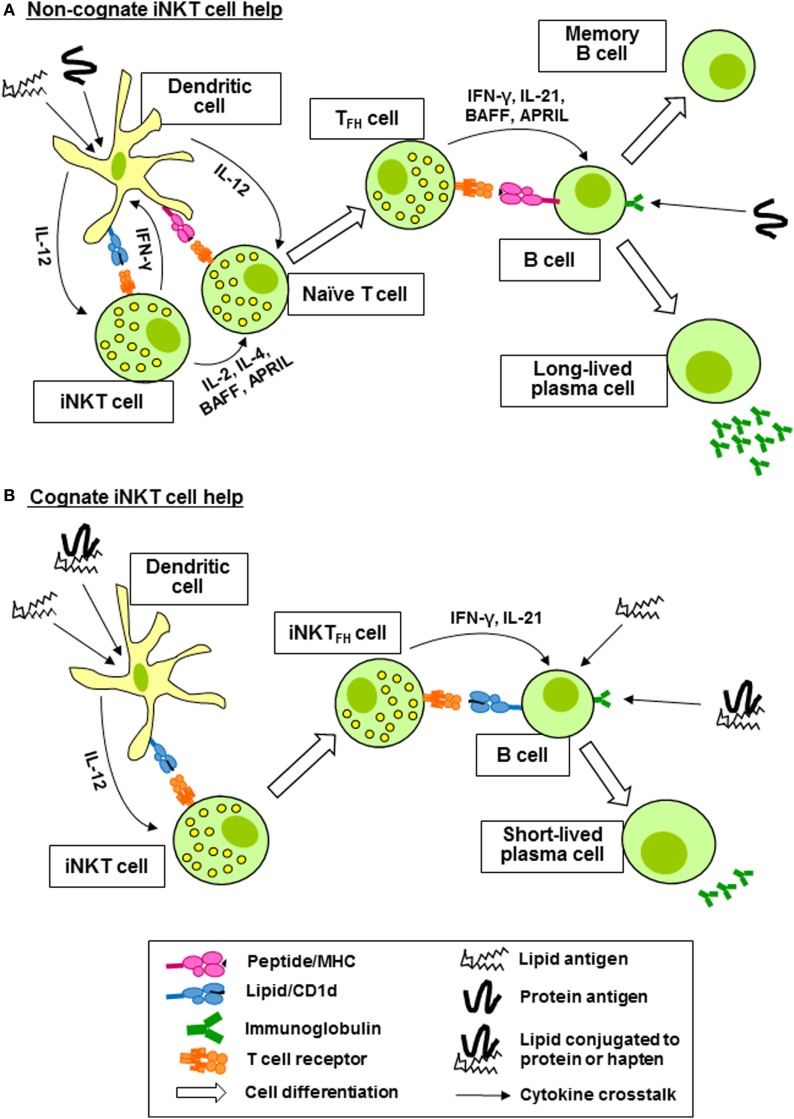
Invariant natural killer T (iNKT) cells provide non-cognate and cognate help for antigen-specific B cells. **(A)** Coadministration of protein antigen and an iNKT cell ligand, such as α-galactosylceramide (lipid antigen) results in internalization by a dendritic cell (DC) and simultaneous presentation of peptide fragments of the protein antigen on major histocompatibility complex (MHC) class II to a naïve CD4^+^ T cell and of the lipid antigen on CD1d to iNKT cells. IFN-γ production by the iNKT cell reciprocally promotes MHC class II antigen presentation and expression of CD40 by the DC, whereas IL-2, IL-4, B cell activation factor (BAFF), and a proliferation-inducing ligand (APRIL) production promotes maturation of the peptide-specific CD4^+^ T cell into a follicular helper T (T_FH_) cell. The T_FH_ cell then provides antigen-specific help for the proliferation of B cells in germinal centers, affinity maturation, antibody class switching, and the generation of long-lived antibody-secreting plasma cells and memory B cells. **(B)** iNKT cells recognizing lipid antigens presented by DC differentiate into follicular helper invariant natural killer T (iNKT_FH_) cells capable of activating B cells specific for lipid antigens or proteins or haptens conjugated to the lipid antigens. B cell activation is mediated by CD40 and CD80/86 ligation by the iNKT_FH_ cells and the production of IL-21 and IFN-γ. Cognate B cell help from iNKT cells results in plasmablast expansion, germinal center formation, modest affinity maturation, and primary class switched antibody production.

Invariant natural killer T cells can activate, regulate, enhance, and sustain humoral immune responses. In the steady state, iNKT cells are distributed throughout the spleen and lymph nodes. Upon activation, they consolidate in the marginal zones of the spleen and the interfollicular regions and medulla of the lymph nodes, where they can interact with APCs and T cells. Later, they are found in the germinal centers (44–48). Coadministration of α-GalCer with immunizing antigen to mice results in enhanced production of antibodies specific for the antigen (49–51). This help provided by iNKT cells is non-cognate and does not require the expression of CD1d by B cells, but requires the co-expression of CD1d and MHC class II by DC and CD40 ligand expression by iNKT cells (52). Upon administration of α-GalCer, iNKT cells residing in the marginal zones of the spleen are activated by CD8α^+^ DC, resulting in reciprocal activation of the DC and their relocation to the borders between the T and B zones of the white pulp where they activate helper T cells specific for the co-administered antigen (Figure 2A). These T cells acquire T_FH_ functions and provide help to cognate B cells (45, 46). The result is a typical thymus-dependent B cell response, with formation of germinal centers, antibody class switching and affinity maturation, and the induction of long-lived antibody-secreting plasma cells and memory B cells (Figure 2A) (46, 51, 53, 54). iNKT cell-associated B cell activation factor (BAFF) and a proliferation-inducing ligand (APRIL) are required for long-term maintenance of the B cell responses (55). Non-cognate help from iNKT cells for the generation of alloreactive antibodies following hepatocyte transplantation in the absence of exogenous glycolipid administration has been demonstrated (56), suggesting that endogenous iNKT cell-activating glycolipids are present. In summary, non-cognate B cell help by iNKT cells boosts adaptive immunity by promoting the generation of long-lived antibody responses and B cell memory.

## iNKT Cells Can Provide Cognate B Cell Help

Invariant natural killer T cells can also provide direct cognate help for B cells reactive against lipid-containing antigens internalized through the BCR (Figure 2B) (57, 58). This has been demonstrated using protein or hapten antigens that are physically linked to α-GalCer, which were shown to be internalized by B cells, leading to presentation of α-GalCer on CD1d molecules and resulting in the acquisition of T_FH_ phenotypes by iNKT cells. This results in reciprocal activation of the B cells, leading to the formation of extrafollicular plasmablasts and germinal centers, affinity maturation, and the generation of robust protein- or hapten-specific immunoglobulin M (IgM) and IgG responses, but not long-lived memory cells (59, 60). Cognate B cell help provided by iNKT cells requires CD1d expression by B cells, CD40-CD40 ligand signaling, CD80-CD86 costimulation, and IFN-γ but not IL-4. The iNKT cells acquire T_FH_ phenotypes, including the expression of Bcl-6 and their ability to provide cognate B cell help requires IL-21 (Figure 2B).

Cognate B cell help by iNKT_FH_ cells that recognize environmental antigens occurs in nature. Mattner and co-workers (61) demonstrated that NKT cells can provide direct cognate help to B cells during infection of mice with *Sphingomonas*, a bacterium that carries iNKT cell agonist lipid antigens in its cell wall. Cognate recognition by iNKT cells of the tumor antigen, *N*-glycolyl-GM3, is also thought to contribute to the generation of specific antibodies, which are present in some people. This antigen binds to CD1d on human B cells and is presented to iNKT cells leading to iNKT cell activation, which may reciprocally drive antibody production by the B cells (62). Sphingolipids that accumulate in Gaucher’s disease are recognized by murine and human type 2 NKT cells that express T_FH_ phenotypes. Injection of mice with these lipids results in NKT_FH_ cell expansion, induction of germinal center B cells, and the production of anti-sphingolipid antibodies. These lipids also activate human NKT cells which provide cognate help to B cells for antibody production *in vitro* (63). Stimulation of iNKT cells isolated from the pleural fluid of humans with tuberculosis with *Mycobacterium tuberculosis* antigens results in their expression of CXCR5, release of IL-21 and the provision of cognate B cell help for the production of IgG and IgA (64). Both T_FH_ and iNKT_FH_ cells contribute to B cell help and the production of antibodies to *Clostridium difficile* toxin B in mice (65). Furthermore, CD1d expression by B cells was required for iNKT cell-mediated B cell help to a protein antigen in mice co-immunized with a mixture of the protein antigen and α-GalCer, indicating that cognate iNKT cell–B cell interactions can play a role in the development of antibody responses to protein antigens (53). With some notable exceptions (66, 67), iNKT_FH_ cells appear to promote weaker antibody responses than conventional T_FH_ cells, with smaller germinal centers and negligible differentiation of long-lived plasma cells and memory B cells (59, 60, 68). Thus, cognate B cell help by iNKT cells boosts innate immunity by promoting the generation of robust, but short-lived, antibody responses to non-protein antigens.

Cognate iNKT cell help for B cell production of antibodies has also been demonstrated unequivocally *in vitro* using co-cultures of primary human B cells and fresh or expanded autologous iNKT cells. Human iNKT cells promote proliferation of autologous naïve and memory B cells and the subsequent production of IgM, IgG, and IgA *in vitro* by a mechanism that requires B cell-iNKT cell contact and CD1d, but not α-GalCer, suggesting that the iNKT cells recognize an autologous ligand (69–71). Surprisingly, antibody blocking experiments suggested that CD40–CD40 ligand interactions were not required for the provision of cognate B cell help by human iNKT cells. Humans have distinct subsets of iNKT cells, including CD4^+^, CD4^−^CD8α^−^β^−^ (double-negative), and CD8α^+^ iNKT cells with distinct but overlapping functional activities (10–12). Separate analysis of the B cell helper activities of CD4^+^, CD8α^+^, and double-negative iNKT cells revealed that all subsets similarly induced B cell proliferation, but CD4^+^ iNKT cells induced higher levels of antibody release (69, 71).

## iNKT Cells Influence B Cell Functions other than Antibody Production

In addition to their roles in antibody production, B cells influence T cell responses. B cells are potent APCs for T cells. They can prime CD4^+^ T cells without the requirement for DC or macrophages (72). Similar to T cells, B cells can produce Th1 and Th2 cytokines (73). Since iNKT cells are unique in that they can selectively secrete Th1, Th2, Th17, or T_reg_ cell cytokines (10, 11, 16, 74), it is possible that these cells may promote and/or regulate cytokine production, antigen presentation, and conventional T cell activation by B cells.

Our group was the first to show that cognate interactions between CD4^+^ iNKT cells and B cells results in the differentiation of B cells into cells with phenotypes of regulatory B (Breg) cells *in vitro* (71). Breg cells are immunosuppressive cells that help maintain immunological tolerance *via* the production of IL-10, IL-35, and transforming growth factor-β (75). Murine B cells expressing CD5 and high levels of CD1d (CD1d^hi^CD5^+^ B cells), mainly marginal zone B cells, secrete IL-10 resulting in immunosuppression and protection against autoimmune disease (76, 77). In humans, a B cell population that produces IL-10 and inhibits Th1 cell responses is present in the CD24^hi^CD38^hi^ B cell compartment, and this subset is functionally impaired in patients with systemic lupus erythematosus (SLE) (78, 79). We found that CD4^+^ human iNKT cells, but not CD8α^+^ or DN iNKT cells, induced the expansion of both CD1d^hi^CD5^+^ and CD24^hi^CD38^hi^ B cells *in vitro* by a mechanism that required cell–cell contact but not activation of the iNKT cells with α-GalCer. Co-culturing CD4^+^ iNKT cells with B cells also induced IL-4 and IL-10 production by the B cells and inhibited their ability to stimulate proliferation of alloreactive and antigen-specific conventional T cells (71). Cognate iNKT cell help by murine iNKT cells has also been shown to induce the expansion of IL-10-producing Breg cells *in vivo*, which was associated with a decrease in germinal center B cell and T_FH_ cell expansion (80). These findings suggest that, as well as promoting antibody production by B cells, CD4^+^ iNKT cells can induce the differentiation of B cells into immunosuppressive cells with impaired ability to present antigen to conventional T cells.

The roles of iNKT cells as promoters and regulators of B cell maturation and antibody responses have important implications for infectious and autoimmune diseases and cancers. In the following sections, we review recent research on the roles of iNKT cells in these classes of diseases, focusing particularly on infectious diseases where antibodies are required for protection, B cell lymphomas and leukemias, and B cell-mediated autoimmune diseases. Finally, we discuss the possible role and treatment potential of iNKT cells in immunodeficiencies that result in impaired or absent antibody responses.

## iNKT Cells Protect Against Infectious Disease

Invariant natural killer T cells play a central role in the protection against infection. A number of bacterial glycolipids have been shown to bind to CD1d and stimulate iNKT cells (7, 8). Administration of α-GalCer prior to pathogen challenge improved disease outcomes in experimental models of infection, including *Plasmodium falciparum, Cryptococcus neoformans, Pseudomonas aeruginosa*, and *M. tuberculosis* (81, 82). Mice lacking CD1d or iNKT cells suffered increase burdens of *Borrelia burgdorferi, Streptococcus pneumonia, P. aeruginosa, M. tuberculosis*, and *Chlamydia pneumonia*.

In murine models, CD1d and iNKT cells are required to generate protective antibody responses against several pathogens, including *P. falciparum* (83), *S. pneumoniae* (84), influenza virus (85, 86), herpes simplex virus (87), *Bacillus anthracis* (88), and *Borrelia* species (89). CD1d and iNKT cells are also needed for the production of IgE antibodies specific for allergens in experimental models of airway inflammation (90, 91) and for the generation of antibodies specific for allo- and xenoantigens in grafted mice (92). Thus, iNKT cells provide an adjuvant activity for the induction of antibody responses. These data suggest that exogenous stimulation of iNKT cells could be used to protect against infection, with glycolipid antigens serving as vaccine adjuvants.

## iNKT Cells Protect Against Cancer

Invariant natural killer T cells are most notable for their roles in antitumor immunity. Mice with deletions in the CD1d or Vα14Jα18 TCR genes, which lack iNKT cells, are predisposed to developing cancer and protection against cancer can be restored by adoptive transfer of iNKT cells (93). Furthermore, glycolipid activation of iNKT cells can both prevent and reverse tumor growth in mice (9, 94). iNKT cells can kill a number of human tumor cell lines *in vitro*, while their activation *in vivo* leads to downstream activation of natural killer (NK) cells and CD8^+^ T cells which infiltrate tumors (95, 96). iNKT cells are deficient and functionally impaired in most human cancers studied (97, 98). These observations have led to a number of clinical trials involving the adoptive transfer of α-GalCer-pulsed autologous DC, *ex vivo* expanded iNKT cells, or both, in cancer patients (99, 100). Although these therapies stimulated antitumor immune responses in the patients, clinical efficacy has to date been limited.

Invariant natural killer T cells play important roles in B cell cancers. Chronic lymphocytic leukemia (CLL), the most common leukemia in adults, is characterized by the expansion of mature monoclonal CD5^+^ B cells (101). These cells can accumulate in the bone marrow and interfere with hematopoiesis, resulting in deficiencies of erythrocytes, platelets, lymphocytes, and antibodies. Since B cells express CD1d, they could potentially prime iNKT cells for cytolysis, one of the cardinal functions of iNKT cells. However, iNKT cells could alternatively provide B cell help for proliferation and antibody production. Recent studies (102–104) have found that circulating iNKT cells are depleted, but functionally active in patients with CLL. Reports are conflicting regarding whether CD1d expression by B cells is higher or lower in CLL patients compared to healthy controls (102, 103, 105–107). Our group provided evidence that downregulation of CD1d expression by CLL cells underlies the functional deficiency of iNKT cells in CLL patients, since the induction of CD1d expression by B cells using retinoic acid restored cytolytic killing of CLL cells by iNKT cells *in vitro* (104). iNKT cells are also depleted from the circulation of patients with multiple myeloma, a malignancy associated with the accumulation of transformed plasma cells in the bone marrow (108, 109). Myeloma cells express CD1d and are sensitive to lysis by NKT cells, but CD1d expression is downregulated during the progression of the disease and eventually lost altogether. iNKT cells are also depleted and functionally impaired in patients with non-Hodgkin’s lymphoma (110) and human herpesvirus 8 multicentric Castleman disease, a virus-induced B cell lymphoproliferative disorder (111). Thus, it appears that iNKT cells play roles in B cell tumor immune surveillance but that these cells become suppressed or depleted during the course of the diseases.

## iNKT Cells Protect Against Autoimmune Disease

Invariant natural killer T cells can also protect against autoimmune and metabolic diseases. Numerical and functional deficiencies of iNKT cells are found in patients with type 1 diabetes (112, 113) and in non-obese diabetic mice, a model of type 1 diabetes (114, 115). Adoptive transfer of iNKT cells from healthy mice protected non-obese diabetic mice from developing diabetes (116). iNKT cells are also depleted from the circulation of patients with multiple sclerosis and they expand in patients during remission (117, 118). They are deficient in mice that are predisposed to developing experimental autoimmune encephalomyelitis (119, 120), and these mice can be protected from developing encephalomyelitis by injection of α-GalCer (121) or by overexpressing the Vα14Jα18 TCR. Patients with SLE (122) and lupus-prone mouse strains (119) have reduced numbers of iNKT cells, suggesting that these cells may protect against lupus. B cell-mediated stimulation of iNKT cells is deficient in patients with SLE, suggesting that B cell defects underlie these iNKT cell deficiencies (123). However, iNKT cells may promote the development of lupus in some animal models. Recent studies have also implicated iNKT cells in obesity and metabolic diseases, including type 2 diabetes (124, 125).

Several lines of evidence suggest that iNKT cells and Breg cells can prevent the production of pathogenic autoantibodies, such as anti-double-stranded DNA IgG antibodies and rheumatoid factor, which are pathogenic in patients with SLE and rheumatoid arthritis, respectively, and in murine models of these diseases (126, 127). Inhibition of autoantibody production is mainly mediated by CD5^+^ marginal zone B cells, which express high levels of CD1d and produce IL-10 in a contact- and CD1d-dependent manner (128, 129). In contrast, iNKT cells can promote non-autoreactive antibody production by follicular B cells *via* the production of IL-17 and IL-21 (128–130). iNKT cells and CD1d also limit autoreactive B cell activation and symptoms of disease in a model where circulating apoptotic cells trigger autoantibody production, resembling the situation in SLE patients (131). Thus, it appears that iNKT cells prevent autoimmunity in lupus-prone mice by inducing autoreactive B cells to differentiate into Breg cells. However, Shen and co-workers (132) showed that iNKT cells promote the production of anti-double-stranded DNA IgG in SLE patients and that expanded iNKT cells from SLE patients, but not healthy donors, induced the production of these autoantibodies by autologous B cells. Therefore, it appears that iNKT cells can either promote or prevent the production of antibodies by B cells and that this balance of activities may determine whether or not an individual develops SLE.

## iNKT Cells and Antibody Deficiencies

Invariant natural killer T cells are also depleted and functionally altered in the circulation of patients with common variable immunodeficiency (CVID), a group of primary antibody deficiencies characterized by recurrent infections and susceptibility to autoimmunity, enteropathy, and lymphoid malignancy (133, 134). Patients with CVID display defects in B cell differentiation, resulting in accumulations of naïve and less-differentiated B cell populations and depletions of class switched memory B cells and plasmablasts (135, 136). This led Erazo-Borrás and co-workers (137) to investigate if the iNKT deficiency underlies the defect in B cell differentiation. They found that, although total iNKT cells were depleted, iNKT_FH_ cells were expanded in the patients. However, α-GalCer-pulsed iNKT cells were unable to induce autologous B cell proliferation although they induced proliferation of healthy donor B cells. These findings suggest that iNKT cells are not impaired, but B responsiveness to iNKT cells is impaired in patients with CVID. Indeed, lipid presentation by B cells is required for the maintenance of iNKT cells (123), suggesting that the defects in B cell differentiation in patients with CVID may lead to the depletions in iNKT cells observed.

## Concluding Remarks

Innate recognition of self and microbial glycolipids enables iNKT cells to drive both cellular and humoral immune responses. Cellular immune responses, as exemplified in antitumor immunity, are mediated in part by the ability of iNKT cells and DC to reciprocally activate each other, resulting in the licensing of DC to activate conventional T cells and NK cells. Similar cognate interactions with B cells contribute to the rapid production of antibodies directed against conserved non-protein antigens resulting in robust, but short-lived, innate humoral immunity. Later, iNKT cells can provide non-cognate help for the generation of antibodies directed against protein antigens, by promoting the maturation and activation of T_FH_ cells, resulting in long-lasting adaptive humoral immunity and the generation of memory B cells. The ability of iNKT cells to promote innate and adaptive humoral immune responses is balanced by their ability to induce maturation of B cells into Breg cells capable of inhibiting the generation of autoantibodies and preventing autoimmune disease. Based on studies in mice, iNKT cells hold great promise as immunomodulators for the treatment of disease. A key challenge with these multifunctional cells will be to harness the protective immunity that they offer for various types of disease while suppressing pathogenic immune stimulation. Several steps have been made to fine-tune iNKT cell stimulation for the treatment of disease. Synthetic glycolipid ligands, including structural analogs of α-GalCer, that bind to CD1d and exhibit partial agonist activity for iNKT cells can increase their therapeutic value while eliminating their pathogenic potential (138–140). Nanoparticle formulations which deliver glycolipid adjuvants to relevant APCs may further enhance their efficacy (141, 142). Tissue-specific modulation of CD1d expression, using epigenetic modifying drugs or retinoic acid, can render cells more susceptible to killing by iNKT cells (104, 143). The ability of iNKT cells to mediate immune protection can also be enhanced using cytokines or by antibody-mediated blocking of immune checkpoint inhibitors, such as PD-1 or cytotoxic T lymphocyte antigen-4 (144). Modification of iNKT cells to express chimeric antigen receptors (CAR-iNKT cells) or homing chemokine receptors may also prove effective for delivering the effector functions of these cells to target organs in the body (145).

## Author Contributions

All authors contributed to the conception, writing, and critical revising of this review.

## Conflict of Interest Statement

The authors declare that the research was conducted in the absence of any commercial or financial relationships that could be construed as a potential conflict of interest.

## Abbreviations

APC, antigen-presenting cell; APRIL, a proliferation-inducing ligand; BAFF, B cell activation factor; Bcl-6, B cell lymphoma-6; BCR, B cell receptor; CAR, chimeric antigen receptor; CLL, chronic lymphocytic leukemia; CVID, common variable immunodeficiency; Breg cell, regulatory B cell; DC, dendritic cell; α-GalCer, α-galactosylceramide; Ig, immunoglobulin; IL, interleukin; iNKT cell, invariant natural killer T cell; iNKT_FH_ cell, follicular helper invariant natural killer T cell; MDSC, myeloid-derived suppressor cell; MHC, major histocompatibility complex; NK cell, natural killer cell; PD-1, programmed death-1; SAP, signaling lymphocytic activation molecule-associated protein; SLE, systemic lupus erythematosus; TCR, T cell receptor; T_FH_ cell, follicular helper T cell; Th cell, helper T cell; T_reg_ cell, regulatory T cell.
